# Estimation of Target Motion Parameters from the Tonal Signals with a Single Hydrophone

**DOI:** 10.3390/s23156881

**Published:** 2023-08-03

**Authors:** Kai Sun, Dazhi Gao, Xiaojing Zhao, Doudou Guo, Wenhua Song, Yuzheng Li

**Affiliations:** 1 Department of Marine Technology, Ocean University of China, Qingdao 266100, China; 2 Department of Physics and Optoelectronic Engineering, Ocean University of China, Qingdao 266100, China

**Keywords:** acoustic field interference, Doppler shift, motion parameter estimation, tonal signals

## Abstract

In the shallow-water waveguide environment, the tonal signals radiated by moving targets carry modal interference and Doppler shift information. The modal interference can be used to obtain the time of the closest point of approach ($t_{CPA}$) and the ratio of the range at the closest point of approach to the velocity of the source ($r_{CPA}/v$). However, parameters $r_{CPA}$ and *v* cannot be solved separately. When $t_{CPA}$ is known, the $r_{CPA}$ and the *v* of the target can be obtained theoretically by using the Doppler information. However, when the Doppler frequency shift is small or at a low signal-to-noise ratio, there will be a strong parametric coupling between $r_{CPA}$ and *v*. In order to solve the above parameter coupling problem, a target motion parameter estimation method from tonal signals with a single hydrophone is proposed in this paper. The method uses the Doppler and modal interference information carried by the tonal signals to obtain two different parametric coupling curves. Then, the parametric coupling curves can be used to estimate the two motion parameters. Simulation experiments verified the rationality of this method. The proposed method was applied to the SWellEx-96 and speedboat experiments, and the estimation errors of the motion parameters were within 10%, which shows the method is effective in its practical applications.

## 1. Introduction

Passive ranging and speed measurement technology of underwater acoustic targets has a wide range of applications in moving target obstacle avoidance, sonar target tracking, underwater early warning, and weapons platforms [1,2,3,4,5]. The estimation of distance can track and monitor other targets and provide position information for underwater weapons platforms. The velocity of the target is another important physical quantity that characterizes the target motion, which can assist in target classification and improve the accuracy of underwater weapons such as torpedo strikes [6,7,8]. In particular, the passive detection technology of a single hydrophone target can be mounted on more flexible platforms, such as buoys and autonomous underwater vehicles (AUVs), to achieve real-time monitoring of large sea areas, which has important applications in marine engineering.

The earliest passive detection techniques include three-subarray ranging [9] and tar-get motion analysis (TMA) [10]. The sound field modeling theory was not mature at this time, so neither method took into account the influence of the shallow-water waveguide environment. Therefore, they performed poorly in shallow seas. In the 1970s, the matching field processing method emerged [11], which is based on the sound field theory model for sound source localization. However, this method has higher requirements for environmental adaptability, and the amount of copy field calculation is large. Therefore, it is difficult to achieve in engineering applications. An important idea for solving the environmental mismatch is to abandon the model and directly extract the target motion parameters from the experimental data. Two of the more representative methods are the acoustic field interference method and Doppler passive detection method.

Broadband noise can form interference fringes of light and dark in shallow-water waveguide environments. In the 1980s, the concept of waveguide invariants was proposed by Chuprov to describe the relationship among frequency, distance, and the slope of interference fringes [12]. The motion parameters contained in the interference fringe can be extracted by using the two-dimensional digital Fourier transform (2D-DFT), the Hough transform, the Radon transform, etc. [13,14,15,16]. In order to solve the coupling problem when using broadband interference fringes to estimate target motion parameters, ref. [17] proposes a motion parameter estimation method combining line spectrum signals and broadband signals. This is effective when the target is closer and is not applicable when the signal-to-noise of broadband signals is relatively low.

However, the radiation noise of ships comprises mainly tonal noise, which has the advantages of a high sound source level and a long propagation distance of a low-frequency sound source. And it has become key in underwater acoustic passive localization technology [18,19]. Rakotonarivo proposed a field differencing method (FDM) to obtain the radial velocity of targets using the tones radiated from a ship [20]. Young et al. [21,22] proposed a maximum likelihood estimator to estimate the waveguide invariant and target distance based on the relationship among the sound intensities of each tone. However, this method requires automatic identification system (AIS) data recorded by other vessels and cannot be applied to distance estimation of noncooperative targets. Next, Chi Jing et al. [23] proposed a target motion parameter estimation method according to the correlation between two resampled tonal signals. However, the method has a coupling problem between motion parameters of the source velocity (*v*) and the range at the closest point of approach ($r_{CPA}$), and the two parameters cannot be separately solved. Parametric coupling means that a bright line appears in the two-dimensional-parameter search result, and the true values of the parameters are on the bright line. However, in practice, it is not possible to determine which point on the coupling line corresponds to the true values of the two parameters, because the cost function on this line has the same value.

Another common way to obtain motion parameters of the target with a tonal signal is to utilize the Doppler shift. Ferguson et al. [24] derived the expression of the Doppler shift during the non-radial motion of the target and used it to estimate the aircraft motion parameters. Next, B. G. Quinn [25] utilized the amplitude estimator to improve the effectiveness of the original algorithm. Xu et al. [26] proposed an exact estimation method for instantaneous frequencies of the MWT transform combined with the Doppler wavelet transform, which was used to estimate the time of the closest point of approach ($t_{CPA}$). In the field of underwater acoustics, Zou et al. [27] proposed the Dopplerlet transform, which can estimate motion parameters of the target and the nearest distance, but needs the target to move faster. Sun et al. [28] proposed a delay-Doppler solution for quadrature operations by the Gram–Schmidt method to obtain the target motion parameters during the radial motion of the target. This method improves the robustness and effectiveness of delay-Doppler parameter estimation. Zou [29] derived the mapping relationship between motion parameters and the Doppler coefficient difference and proposed a passive estimation method based on the latter. This can estimate the motion parameters of near-field targets according to the least squares method. In 2021, the Doppler-warping transformation was proposed by Gao, which uses the Doppler frequency shift of the tonal signals, which can be used to estimate the speed of the sound source under the premise of a known distance [30]. However, in the case of a small Doppler frequency shift or low signal-to-noise ratio, there is a serious parameter coupling problem between *v* and $r_{CPA}$, so that it is impossible to accurately estimate the two parameters.

In previous studies, when estimating the target motion parameters *v* and $r_{CPA}$ from the tonal signals with a single hydrophone, the coupling phenomenon of the two parameters has been very serious, so that the two parameter values could not be given separately. In order to solve the above parametric coupling problem, a target motion parameters estimation method combined with interference information and Doppler frequency shift information from two tones is proposed in this paper. The proposed method does not require hydrophone arrays or broadband noise signals, and only uses two or more tonal signals received by one single hydrophone from the same target to achieve the estimation of motion parameters *v* and $r_{CPA}$. The proposed method makes full use of the information carried by two or more tones, and can be used for the estimation of parameters $r_{CPA}$ and *v* in the case of a small Doppler frequency shift and low signal-to-noise ratio. It is worth emphasizing that the method proposed in this paper does not require detailed environmental parameters except for the waveguide invariant and local sound speed value. Moreover, the method proposed in this article does not require the target to be a cooperative target. In addition, in most shallow-sea waveguide environments, when the environmental parameters are unknown, the waveguide invariant value can be set to 1, the sound speed value in the water is 1500 m/s, and the final parameter estimation error generally does not exceed 25%.

This article is organized as follows: the parameter searching algorithm is introduced in Section 2. In order to validate the proposed method, a simulation, method performance analysis and two experiments are given in Section 3. Finally, a conclusion is given in Section 4.

## 2. Methods to Estimate the Motion Parameters

### 2.1. Sound Field Interference Theory

In the shallow-water waveguide environment, the acoustic source is located at depth $z_{s}$, the hydrophone is located at depth *z*, and the received sound signal can be expanded into a simple positive wave superposition form:

(1) $$p(r,\omega )=A(\omega )\sum_{n=1}^{N}B_{n}(z,z_{s})\exp\left[-i(\omega t-k_{n}r+\pi /4)\right]$$

where $B_{n}(z,z_{s})={\left(2\pi /k_{n}r\right)}^{\frac{1}{2}}\Psi_{n}(z)\Psi_{n}(z_{s})$, $\Psi_{n}$ is the nth number of normal mode vertical distribution functions, $k_{n}$ is the horizontal wavenumber of the nth normal mode, and r is the horizontal distance between the acoustic source and the hydrophone. According to the sound pressure expression, the sound intensity expression is obtained, as shown in Equation (2).

(2) $$I(r,\omega )=A^{2}(\omega )\left\{\sum_{n}B_{n}{}^{2}+2\sum_{\begin{array}{l} n,m\\ n\neq m \end{array}}B_{n}B_{m}\cos\left[\Delta k_{nm}(\omega )r\right]\right\}$$

The first item in parentheses in Equation (2) is a noninterfering term, and the second item is an interfering term. For the same interference fringe at different frequencies, distances meet the second term equality; thus, Equation (3) is established.

(3) $$\Delta k_{nm}(\omega_{1})r_{1}=\Delta k_{nm}(\omega_{2})r_{2}$$

$\Delta k_{nm}$ can be expressed in the following form [31]:

(4) $$\Delta k_{nm}(\omega )=(\gamma_{n}-\gamma_{m})\omega^{-\frac{1}{\beta }}=\gamma_{nm}\omega^{-\frac{1}{\beta }}$$

Substitution of Equation (4) into Equation (3) yields Equation (5).

(5) $$\frac{r_{1}}{r_{2}}={\left(\frac{\omega_{1}}{\omega_{2}}\right)}^{\frac{1}{\beta }}$$

For the same interference fringe, Equation (6) holds.

(6) $$I(r_{2},\omega_{2})=\frac{A^{2}(\omega_{2})}{A^{2}(\omega_{1})}I\left(r_{2}{\left(\frac{\omega_{1}}{\omega_{2}}\right)}^{\frac{1}{\beta }},\omega_{1}\right)$$

**Figure 1:**
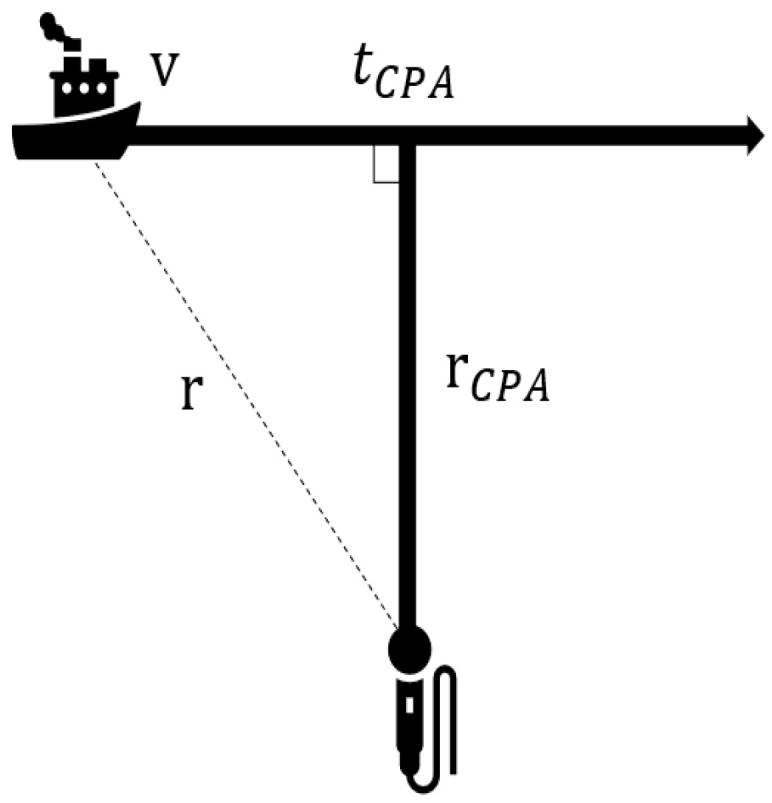
Schematic of the hydrophone position and target motion.

The motion trajectories of the hydrophone and acoustic source in the motion model of this paper are not collinear, as shown in Figure 1, and the relationship between distance *r* and time *t* is shown in Equation (7).

(7) $$r=\sqrt{{\left[v(t-t_{CPA})\right]}^{2}+r_{CPA}{}^{2}}$$

Document 23 defines a new time domain based on this situation [23].

(8) $$t^{r}=\frac{r}{v}=\sqrt{{\left(t-t_{CPA}\right)}^{2}+b^{2}}$$

where $b=\frac{r_{CPA}}{v}$ and the new time given by Equation (8) domain are proportional to distance *r*. The following Equation holds.

(9) $$I(t_{2}{}^{r},\omega_{2})=\frac{A^{2}(\omega_{2})}{A^{2}(\omega_{1})}I\left(t_{2}{}^{r}{\left(\frac{\omega_{1}}{\omega_{2}}\right)}^{\frac{1}{\beta }},\omega_{1}\right)$$

The above Equation reveals that $I(t_{2}{}^{r},\omega_{2})$ is proportional to $I\left(t_{2}{}^{r}{\left(\frac{\omega_{1}}{\omega_{2}}\right)}^{\frac{1}{\beta }},\omega_{1}\right)$ in the new time domain and that $t^{r}$ contains motion parameter information. Thus, the cost function can be set according to Equation (9), and the target motion parameter *b*, $t_{CPA}$ can be obtained [23].

### 2.2. Doppler Shift and Doppler-Warping Transformation

The tonal noise radiated from ships is not only interferometric noise but also has a Doppler shift. The acoustic source moves at uniform velocity *v* on one side of the hydrophone (as shown in Figure 1), and due to the radial velocity relative to the hydrophone, a Doppler shift is generated. This makes the frequency of the signal received by the hydrophone vary with time. This change is reflected in the frequency spectrum of the received signal by changing from a single frequency to a narrow band.

Ref. [23] shows the relationship between instantaneous frequencies and time when the acoustic source is moving and the hydrophone is stationary, where $f_{0}$ is the original frequency of the acoustic source, *c* is the sound speed of the water body, *v* is the motion speed of the acoustic source, $r_{CPA}$ is the range at the closest point of approach, $\tau_{0}$ is the time when the acoustic source reaches the nearest point, and t is the signal reception time.

(10) $$f(t)=f_{0}\frac{c^{2}}{c^{2}-v^{2}}\left[1-\frac{v^{2}(t-\tau_{0})}{\sqrt{r_{CPA}{}^{2}(c^{2}-v^{2})+c^{2}v^{2}{\left(t-\tau_{0}\right)}^{2}}}\right]$$

According to the target motion model in Figure 1, there is a relationship between distance *r* and time *τ*, as shown in Equation (11), where *τ* is the time at which the signal is emitted.

(11) $$r=\sqrt{{\left[(\tau -\tau_{0})v\right]}^{2}+r_{CPA}{}^{2}}$$

It is assumed that the acoustic source transmits a tonal signal, as shown in Equation (12), and that only forward propagation exists during the propagation of sound waves. Therefore, the signal is expressed as Equation (13), where *t* is the time the signal is received by the hydrophone, and k=ω/c is the horizontal wavenumber.

(12) p(τ)=exp(jωτ)

(13) $$p(t,r)=B(t)\times \exp\left[j(\omega t-kr)\right]$$

Substitution of Equation (11) into Equation (13) yields Equation (14).

(14) $$P=A\times \exp\left\{j\omega \left[t-\frac{\sqrt{{\left((\tau -\tau_{0})v\right)}^{2}+r_{CPA}{}^{2}}}{c}\right]\right\}$$

The acoustic signal is transmitted at time *τ* and received by the hydrophone at time *t*. The sound wave phases of both time points should be equal, so Equation (15) holds.

(15) $$\tau =t-\frac{\sqrt{\left[(\tau -\tau_{0})v^{2}\right]+r_{CPA}{}^{2}}}{c}$$

Because the acoustic signal emission time is *τ* and the receiving time is *t*, *t > τ*, Equation (15) can be sorted to obtain Equation (16).

(16) $$\tau =\frac{c^{2}t-v^{2}\tau_{0}-\sqrt{r_{CPA}{}^{2}(c^{2}-v^{2})+c^{2}v^{2}{\left(t-\tau_{0}\right)}^{2}}}{c^{2}-v^{2}}$$

Substitution Equation (16) into the source signal expression Equation (12) yields Equation (17), which is the expression of receiving the signal by a hydrophone.

(17) $$p(t)=B(t)\times \exp\left[j\omega \frac{c^{2}t-v^{2}\tau_{0}-\sqrt{r_{CPA}{}^{2}(c^{2}-v^{2})+c^{2}v^{2}{\left(t-\tau_{0}\right)}^{2}}}{c^{2}-v^{2}}\right]$$

where *B(t)* is the amplitude of the receiving signal. The expression of the instantaneous frequency is Equation (10), which can be derived from the derivative of the phase of the signal with respect to *t*. Equation (17) shows that the phase of the receiving signal changes to nonlinear due to the presence of a Doppler effect.

The basic idea of the warping transformation is to take the inverse function of the instantaneous phase of the signal as the time domain warping operator and then use the warping operator to resample the signal so that the nonlinear phase of the signal becomes a linear phase. According to Equation (15), the Doppler-warping operator can be set as shown in Equation (18), which contains the parameters $\tau_{0}$, v, $r_{CPA}$, and c [30].

(18) $$h(t)=t+\frac{\sqrt{{\left[(t-\tau_{0})v\right]}^{2}+r_{CPA}{}^{2}}}{c}$$

When all the parameters in the Doppler-warping operator are correct, by substituting Equation (18) into Equation (17), we obtain Equation (19).

(19) P(t)=B(t)×exp(jωt)

Equation (19) indicates that when all parameters are correctly inputted, the nonlinear phase of the received signal can be restored to a linear phase by the Doppler-warping transformation. The phase of Equation (19) is differentiated with respect to time *t* to obtain the instantaneous frequency of $f(t)=f_{0}$, and the instantaneous frequency change due to the Doppler is eliminated by the Doppler-warping transformation. This change in the signal spectrum is from a narrowband signal to a single frequency, and the spectral entropy decreases, so the spectral entropy can be applied as a cost function. When the Doppler frequency shift is small or at a low signal-to-noise ratio, using the Doppler frequency shift to estimate the target motion parameters *v* and $r_{CPA}$ also has a strong parameter coupling phenomenon. This results in the inability to directly estimate the two parameters using the Doppler information.

### 2.3. Target Motion Parameter Estimation Step

To solve the parameter coupling problem when estimating motion parameters *v* and $r_{CPA}$, this paper proposes a method for the combination of Doppler frequency shift and interference, which fully utilizes the information carried by the ship’s radiation tones. In this paper, the Doppler parameter coupling curve and the two tones interferometry parameter ratio line are obtained, the two coupling lines intersect, and the intersection point is the final estimation result. The proposed method is carried out in three steps as follows:

The first step is the Doppler-warping transformation [30].

1. Select one of two similar frequency signals with Doppler shifts in the received signal low-frequency analysis recording (LOFAR) plot and select the upper and lower limit frequencies of the tones.
2. The speed of sound in water *c* is known; the search grid *v* = [*v_1_, v_2_,..., v_n_*] and $r_{CPA}$ = [*r_1_, r_2_,..., r_m_*], where n and m are the *v* and $r_{CPA}$ search grid dimensions, respectively.
3. Select the (*v*,$r_{CPA}$) combinations in the search grid and use the Doppler-warping operator (Equation (18)) to resample the original signal.
4. The fast Fourier transform (FFT) algorithm is used for the resampled signal to obtain the spectrum $x_{p}(f)$. Select the upper and lower limits of the frequency band $f_{1},f_{2}$ to be analyzed and calculate the spectral entropy function *S_D_* within the frequency band.
  (20) $$S_{D}(v_{n},r_{m})=-\int_{f_{1}}^{f^{2}}X_{p}(f)\log_{2}X_{p}(f)df$$
5. Repeat steps (3) and (4) until the full *v*, $r_{CPA}$ mesh search is completed.
6. The two-dimensional color plot is drawn according to the cost function *S_D_*, and the minimum value of $S_{D}(:,j)$ searched at each $r_{CPA}$ grid is selected. These values are employed in the triple polynomial fit to obtain the parametric coupling curve.

The second step, using two tones interference, extracts the motion parameters [23].

1. The (*v*,$r_{CPA}$) combination that minimizes the Doppler-warping transformation cost function value is selected to resample the original signal to obtain a signal without a Doppler shift.
2. Calculate the LOFAR plot of the signal and select the intensities of two tones $I(t,\omega_{1})$ and $I(t,\omega_{2})$ that are excited by the same acoustic source.
3. Set up the search grid $b=[b_{1},b_{2}\ldots b_{n}]$, where $b=\frac{r_{CPA}}{v}$.
4. Select the mesh parameters that convert *t* to $t^{r}$ according to Equation (8), resample the two tones in the $t^{r}$ domain, and calculate the cost function $S_{2}$, where $t_{1}{}^{r}$ and $t_{2}{}^{r}$ are the upper limit and lower limit, respectively, after the coordinate conversion.
  (21) $$S_{2}(b_{m})=\frac{\int_{t_{1}{}^{r}}^{t_{2}{}^{r}}{I^{\prime }}_{r}\left[\omega_{1},t^{r}(b_{m})\right]I^{\prime }\left[\omega_{2},t^{r}(b_{m}){\left(\frac{\omega_{2}}{\omega_{1}}\right)}^{\frac{1}{\beta }}\right]dt^{r}}{\sqrt{\int_{t_{1}{}^{r}}^{t_{2}{}^{r}}{\left|{I^{\prime }}_{r}\left[\omega_{1},t^{r}(b_{m})\right]\right|}^{2}dt^{r}\int_{t_{1}{}^{r}}^{t_{2}{}^{r}}{\left|I^{\prime }\left[\omega_{2},t^{r}(b_{m}){\left(\frac{\omega_{2}}{\omega_{1}}\right)}^{\frac{1}{\beta }}\right]\right|}^{2}dt^{r}}}$$
5. Repeat steps (3) and (4) until all grid searches are complete.
6. The b corresponding to the peak of the cost function is the final estimation result.

In the third step, the Doppler parameter coupling curve obtained in the first step is intersected with the straight line $r_{CPA}/v$ obtained in the second step, and the corresponding parameter value of the intersection point is the estimated *v* and $r_{CPA}$.

## 3. Results

### 3.1. Simulation Results

#### 3.1.1. Theoretical Simulation

**Figure 2:**
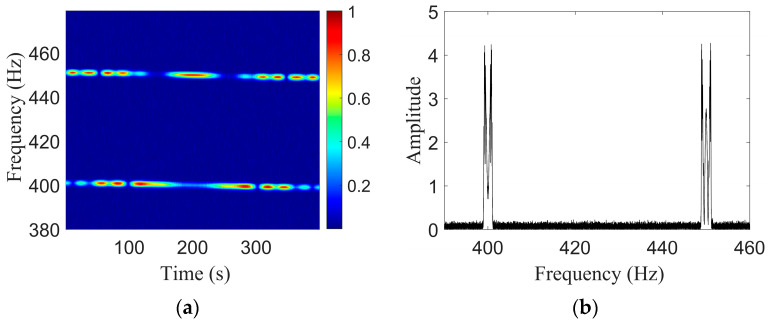

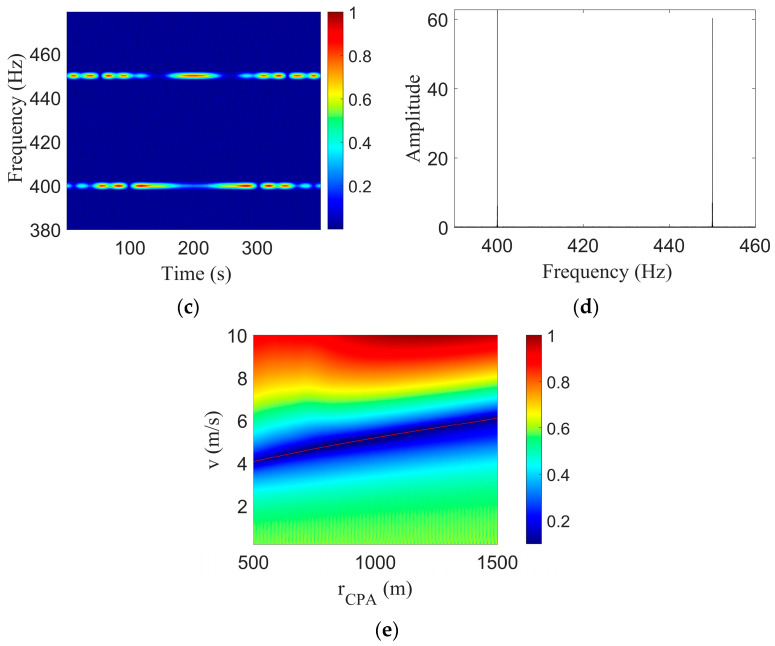
Doppler-warping transformation simulation results. (**a**) LOFARgram of the signal; (**b**) The FFT amplitude spectrum of the signal; (**c**) LOFARgram of the signal without Doppler; (**d**) The FFT amplitude spectrum of the signal without Doppler; (**e**) Doppler-warping transformation results.

Shallow-sea acoustic field simulation parameters are listed as follows: a 200 m range-independent Pekeris waveguide, sound speed of the water body of 1500 m/s, seafloor sound speed of 1593 m/s, density of 1.76 ${g/\mathrm{cm}}^{3}$, and seafloor attenuation coefficient of 0.2 dB/λ. The acoustic source transmits two tonal signals of 400 Hz and 450 Hz, and moves in a straight line at a speed of 5 m/s at a depth of 50 m. The range at the closest point of approach is $r_{CPA}$ = 900 m; the time of the closest point of approach is $t_{CPA}$ = 200 s; and the hydrophone placement depth is 100 m. The signal-to-noise ratio of the received signal is 6 dB, and the total signal duration is 400 s. The LORAF of the received signal is shown in Figure 2a, which shows that the instantaneous frequency changes with time, and the corresponding signal spectrum is that shown in Figure 2b. It can be seen that the frequency band widening phenomenon caused by the Doppler effect exists at the center frequencies of 400 Hz and 450 Hz.

When the Doppler-warping operator parameters are correct, the LOFARgram of the signal changes from Figure 2a–c, and the instantaneous frequency that originally changes with time becomes a single frequency. The corresponding spectrogram changes from Figure 2b–d, and the original wider frequency band caused by the Doppler effect becomes a single frequency band. The Doppler-warping transformation search results are shown in Figure 2e, where the search grids are v=[0.2:0.2:10] and $r_{CPA}=[500:5:1500]$. The blue region in Figure 2e corresponds to the minimum spectral entropy results, and there is a strong parameter coupling phenomenon after the Doppler-warping search. The red curve in the figure is the parametric coupling curve, which is obtained by using a cubic polynomial fitting.

The combination $\left(v,r_{CPA}\right)$ that can yield the lowest cost function is selected to resample the original signal to obtain a signal without a Doppler shift. After resampling the signal, STFT is performed to obtain the signal spectrogram. The STFT window length is 1 s, the step length is 0.1 s, and the window type is Hanning. Two tonal signals of 400 Hz and 450 Hz are selected in the LOFARgram, and the second step is to extract the target motion parameters according to the two tones interference, where β=0.98 and the search grid is b=[100:1:220].

**Figure 3:**
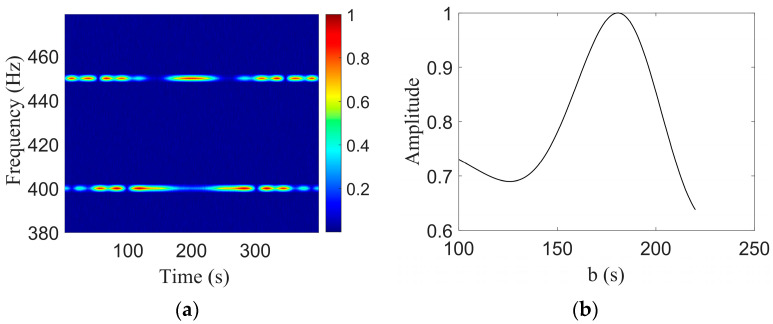
Two-tone interference simulation results. (**a**) LOFARgram of the signal; (**b**) Search results of b.

Figure 3b is a graph of the parameter b search results, and the peak position corresponds to the estimate $\hat{b}=181s$ and the true value b=180s; both are similar. As $b=r_{CPA}/v$, a straight line with a ratio of v to $r_{CPA}$ is obtained.

**Figure 4:**
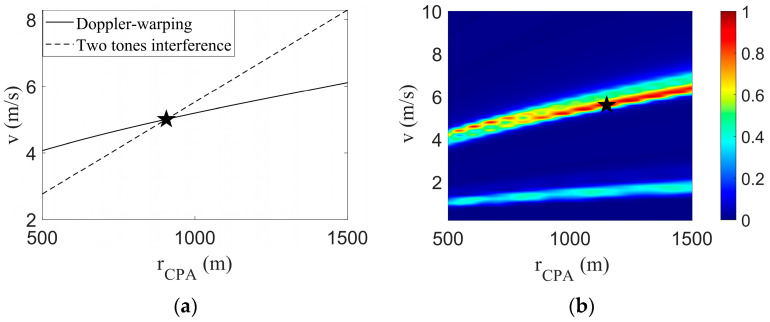
Federated search results. And the black star represents the position of the parameters estimated by the algorithm. (**a**) This article method search result; (**b**) FDM search results.

By combining the parametric coupling curve obtained by the Doppler-warping transformation with the parametric coupling line obtained by the two tones interference, Figure 4 is obtained.

The Doppler parameter coupling curve intersects with the interference parameter coupling line, and the corresponding *v* and $r_{CPA}$ of the intersection points of the two lines are the final estimation results, as shown in the black five-pointed star in Figure 4a. The estimated results were $\hat{v}=5.01\;m/s$ and ${\hat{r}}_{CPA}=906.1\;m$; the target true parameter values were v=5.00 m/s and $r_{CPA}=900.0\;m$; the *v* estimation error was 0.2%; and the $r_{CPA}$ estimation error was 0.7%. A simulation experiment showed the rationality of the method.

In the condition of unknown environmental parameters, the waveguide invariant value was set to 1, the sound speed of the water body was 1500 m/s, and the estimation errors of parameters *v* and $r_{CPA}$ were 1.2% and 3.3%, respectively.

The field differencing method (FDM) algorithm search results are shown in Figure 4b, where the black pentagram represents the final search results $\hat{v}=5.6\;m/s$, ${\hat{r}}_{CPA}=1150\;m$. The estimation errors of the two parameters were 12% and 28%, respectively. The parametric coupling problem can be obviously seen in Figure 4b. There are three main reasons for the large errors and parametric coupling problem: (1) the signal duration is shorter, and the longer the FDM method, the more accurate the search results. (2) Around $t_{CPA}$, the radial velocity changes rapidly, resulting in a large calculation error of the FDM algorithm. (3) The presence of the Doppler shift leads to inaccurate frequency extraction.

#### 3.1.2. Method Performance Analysis

In this section, the influence of the signal-to-noise ratio (SNR), the sound speed c of the water body, and the waveguide invariants *β* on the methods proposed in this paper will be discussed.

**Figure 5:**
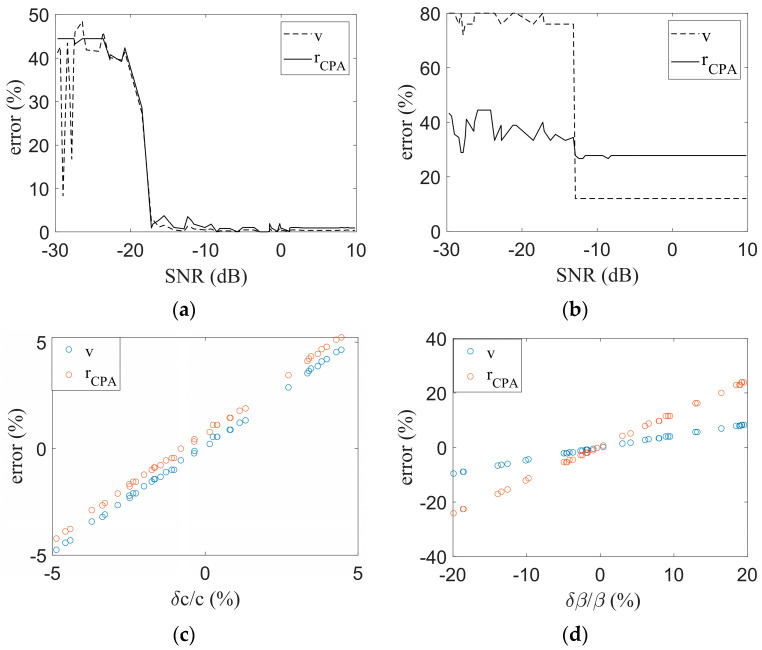
Influence of different parameters on the methods. (**a**) The influence of SNR on the method proposed in this paper; (**b**) The influence of SNR on FDM; (**c**)The influence of c on the method proposed in this paper; (**d**) The influence of β on the method proposed in this paper.

Figure 5a shows the influence of the signal-to-noise ratio on the performance of the method. The signal-to-noise ratio was set to be randomly distributed between −30 dB and 10 dB, the noise type is Gaussian white noise, and the power signal-to-noise ratio was applied. As is shown in Figure 5a, the method proposed in this paper has a certain robustness for Gaussian white noise, the method performance is better when the signal-to-noise ratio is higher than −18 dB, and the method performance begins to gradually deteriorate when the signal-to-noise ratio is lower than −18 dB.

Compared to Figure 5b, the method proposed in this paper is at least 5 dB more robust to noise than FDM. In the condition of low SNR, the proposed method has better performance mainly in the following three aspects: 1. The simulation in this paper adopts the definition of power SNR, although the overall SNR is relatively low, but the SNR at the frequency point where the tonal signal is located is relatively high. 2. This paper adopted the method of eliminating the Doppler frequency shift first, so that the acoustic signal energy was relatively concentrated. 3. Compared with FDM, the proposed method uses the whole signal for analysis, rather than only using some of the points, so it has better robustness for Gaussian white noise.

Figure 5c shows the impact of sound speed error on the performance of the method. In actual applications, the sound speed can be obtained from preliminary measurements. Therefore, the sound speed error is not too large; this study set it within a random distribution between −5% and 5%. Figure 5c shows that the effect of sound speed on the two parameters was similar. This finding can be explained by the notion that the sound speed error of the water body affects the Doppler-warping result, and the two-tone interference does not involve the parameter of the sound speed of the water body. Therefore, the two tone interference result will not have an impact, and (r+Δr)/(v+Δv)=r/v=const, where Δr and Δv are the errors. As Δr/r=Δv/v, the two parameter errors undergo the same changes. However, there is a DC component of the two parameter errors because the inaccurate waveguide invariant or signal-to-noise ratio in the simulation leads to inaccurate estimation.

Figure 5d shows the influence of waveguide invariants on the proposed method. The other parameters remain unchanged when exploring the influence of waveguide invariants. In this study, the waveguide invariant error was randomly distributed between −20% and 20%. When the waveguide invariant *β* changes, according to Equation (8), the relationship between the parameter *b* and the waveguide invariant *β* can be obtained, as shown in Equation (22).

(22) $$b=\sqrt{{\left(t_{2}^{r}\right)}^{2}{\left(\frac{\omega_{1}}{\omega_{2}}\right)}^{\frac{2}{\beta }}-{\left(t_{CPA}-t\right)}^{2}}$$

Equation (22) is the *β* variation in parameter *b*, and *β* varies around 1 in general waveguide environment. Therefore, Equation (22) can be written as shown in Equation (23).

(23) $$\delta b={\left.\delta \beta \frac{\partial b}{\partial \beta }\right|}_{\beta =1}=\frac{\delta \beta {\left(t_{2}^{r}\right)}^{2}{\left(\frac{\omega_{1}}{\omega_{2}}\right)}^{2}\ln\frac{\omega_{1}}{\omega_{2}}}{\sqrt{{\left(\frac{\omega_{1}}{\omega_{2}}\right)}^{2}{\left(t_{2}^{r}\right)}^{2}-{\left(t_{CPA}-t\right)}^{2}}}$$

Since $b=r_{CPA}/v$, δb can be written as shown in Equation (24).

(24) $$\delta b={\left.\frac{\partial b}{\partial r_{CPA}}\delta r_{CPA}\right|}_{\begin{array}{l} r_{CPA}=r_{0}\\ v=v_{0} \end{array}}+{\left.\frac{\partial b}{\partial v}\delta v\right|}_{\begin{array}{l} r_{CPA}=r_{0}\\ v=v_{0} \end{array}}=\frac{1}{v_{0}}\delta r_{CPA}-\frac{r_{0}}{v_{0}{}^{2}}\delta v$$

In Equation (24), *r*_0_ and *v*_0_ represent the true motion parameter values.

When the waveguide invariant *β* changes, the parameter *c* is considered to be constant, so the Doppler parameter coupling curve does not change. Near the true point of the Doppler parameter coupling curve, it can be approximated that the two parameters are linear. Thus Equation (25) holds.

(25) $$\delta v=k\times \delta r_{CPA}$$

Combining Equation (23), Equation (24), and Equation (25) together, Equation (26) was obtained.

(26) $$\delta r_{CPA}=\delta \beta \frac{{\left(t_{2}^{r}\right)}^{2}{\left(\frac{\omega_{1}}{\omega_{2}}\right)}^{2}\ln\frac{\omega_{1}}{\omega_{2}}}{\sqrt{{\left(\frac{\omega_{1}}{\omega_{2}}\right)}^{2}{\left(t_{2}^{r}\right)}^{2}-{\left(t_{CPA}-t\right)}^{2}}}\frac{v_{0}^{2}}{v_{0}-kr_{0}}$$

When there is an error in the β, the estimation error of the motion parameter is related to the frequency of the tonal signals, the true parameter value, and the slope of the Doppler parameter coupling curve near the true parameter values. When the δβ is small, the estimation error of the parameter is approximately linear with the δβ.

### 3.2. Experimental Results and Analysis

The method proposed in this paper was applied in two offshore experiments to show its effectiveness. The two experiments were the Swellex-96 experiment conducted by the Scripps Institute of Oceanography of the University of California in May 1996 and the speedboat experiment conducted by the Hydroacoustic Department of the Ocean University of China in June 2021, in offshore Qingdao.

#### 3.2.1. SwellEx-96 Experiment

**Figure 6:**
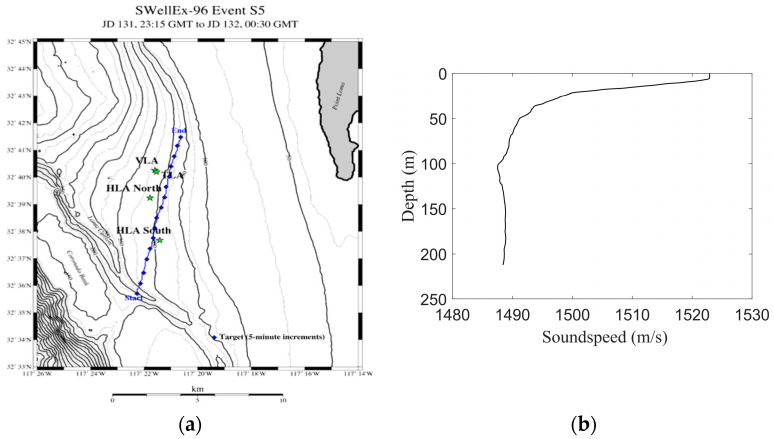
Event S5 condition. (**a**) Source trajectory and array position; (**b**) Sound speed of the water body.

The SwellEx-96 experiment was conducted by the University of California, Scripps Institute of Oceanography, on 10–18 May 1996, approximately 12 km from the tip of Point Loma near San Diego, California. The experiment used two drag acoustic sources to transmit multitone signals: a shallow-water (9 m) acoustic source and a deep-water (54 m) acoustic source. The acoustic sensors deployed in the experiment included a vertical line array (VLA), a tilted line array (TLA), and two horizontal line arrays (HLAs) installed on the seabed. The experiments were divided into Event S5 and Event S59, and Event S5 was selected for analysis in this paper. The acoustic source motion and sensor deployment position in Event S5 are shown in Figure 6a, and the blue polyline is the acoustic source motion trajectory. The sound speed of the water body in the experimental sea area is shown in Figure 6b, and the average sound speed was 1488 m/s.

**Figure 7:**
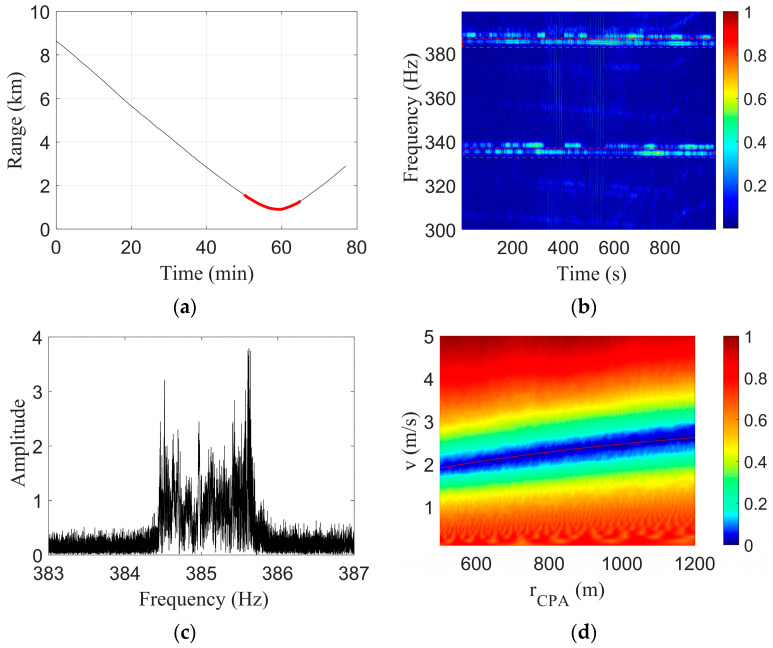
Results of the two-tone interference in the speedboat experiment. (**a**) LOFARgram of the signal without a Doppler shift; (**b**) Search results for b; (**c**) Spectrum of the 385 Hz tonal signal; (**d**) Doppler-warping transformation result.

In this study, the shallow-water acoustic source data recorded by VLA array 13 were selected for analysis. Array No. 13 is located at a depth of approximately 150 m; the range at the closest point of approach from the acoustic source to the hydrophone is $r_{CPA}=903\;m$; and the time of the closest point of approach is $t_{CPA}=3540\;s$. The 3000–4000 s data recorded by the 13th array element, represented by the red curve shown in Figure 7a, were selected for analysis. According to the global positioning system (GPS) data, the average motion speed of the acoustic source in the selected time period was v=2.38 m/s, so the ratio of the range at the closest point of approach to the speed was $b=r_{CPA}/v=379\;s$. In this study, the two tonal signals of 335 Hz and 385 Hz transmitted by a shallow-water acoustic source in Event S5 were selected for analysis. The spectrogram of the selected signal is shown in Figure 7b, and the analyzed frequencies in this article are marked with red dotted lines in Figure 7b.

In this study, the 385 Hz tonal signal was selected for the Doppler-warping transformation, the signal spectrum is shown in Figure 7c, and the signal band was widened by the Doppler effect. The search grids were v=[0.1:0.1:5] and $r_{CPA}=[500:10:1200]$. The average sound speed of c=1488 m/s was chosen. The Doppler-warping transformation result is shown in Figure 7d, where the red curve is the parametric coupling curve obtained by cubic polynomial fitting.

**Figure 8:**
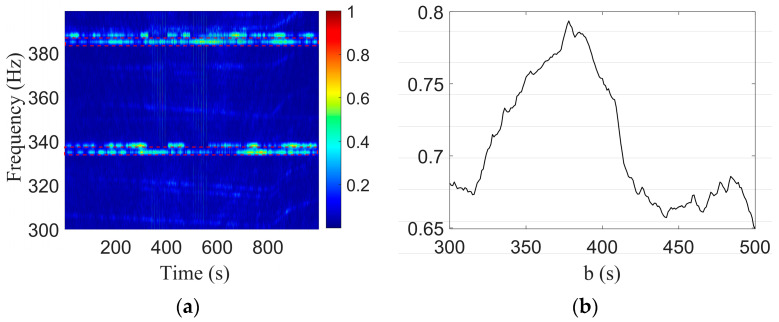
Results of two-tone interference of Event S5. (**a**) LOFARgram of the signal without a Doppler shift; (**b**) Search results of b.

The parameter combination (2.6,1145) corresponding to the minimum value of the Doppler-warping cost function was selected and used to resample the original signal to eliminate the Doppler shift. The LOFAR of the signal without a Doppler shift obtained by the STFT is shown in Figure 8a. The STFT time window length was 1 s, the step length was 0.1 s, and the time window type was the Hanning window.

**Figure 9:**
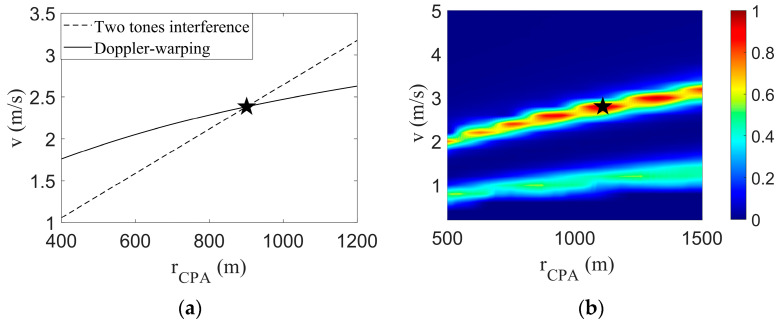
Federated search results of Event S5. And the black star represents the position of the parameters estimated by the algorithm. (**a**) This article method search results; (**b**) FDM search results.

The two tones are marked by the red dashed lines in Figure 8a. The parameter b was estimated according to the modal interference information, where the $t^{r}$ interval was 0.05 s and the waveguide invariant was β=0.92. The one-parameter search grid was set to b=[300:1:500]. Figure 8b was obtained, and the abscissa corresponding to the peak in the figure was the final estimated result $\hat{b}=378s$. Since $b=r_{CPA}/v$, a straight line about the relationship between *v* and $r_{CPA}$ was obtained according to b. This line was combined with the parametric coupling curve obtained by Doppler-warping transformation, as shown in Figure 9.

Figure 9a is the final result graph, the position of the pentagram is the intersection of two coupling lines, and its corresponding coordinate values are the final parameter estimates $\hat{v}=2.384\;m/s$ and ${\hat{r}}_{CPA}=901\;m$. The true values of the parameters were v=2.38 m/s and $r_{CPA}=903\;m$, and their estimation errors were 0.17% and 0.22%, respectively. Both values were less than 5%, which verifies the effectiveness of the method.

In the condition of unknown environmental parameters, the waveguide invariant value was set to 1, the sound speed of the water body was 1500 m/s, and the estimation errors of parameters *v* and $r_{CPA}$ were 5% and 12%, respectively.

The FDM algorithm search results are shown in Figure 9b, where the black pentagram represents the final search results $\hat{v}=2.8\;m/s$ and ${\hat{r}}_{CPA}=1110\;m$. The estimation errors of the two parameters were 18% and 23%, respectively. The phenomenon of parameter coupling can also be seen in Figure 9b.

#### 3.2.2. Analysis of the Speedboat Experiment

**Figure 10:**
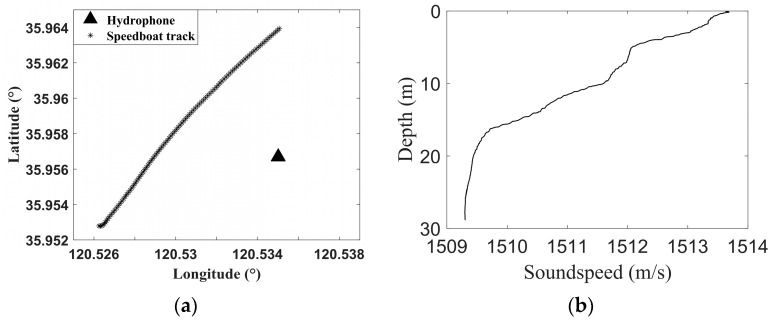
Speedboat track, hydrophone position, and sound speed of the water body. (**a**) Speedboat track and hydrophone position; (**b**) Sound speed of the water body.

In June 2021, a hydroacoustic experiment was conducted at the Department of Hydroacoustics at the Ocean University of China in the coastal waters of Qingdao, which employed the icListen hydrophone produced by Ocean Sonics in Canada with a sampling rate of 128 kHz. This was anchored by the receiving ship, which was lowered to 5 m below the water’s surface. The target ship of the experiment was a speedboat, and the acoustic source was the tonal noise emitted by the speedboat. Both the speedboat and the receiving boat were equipped with a differential GPS, which recorded the location information in time. During the experimental period, which was the fishing ban period in the Yellow Sea, there were no vessels near the site, with the exception of the target ships. The trajectory of the speedboat is shown in Figure 10a. The sound speed of the water body in the experimental sea area is shown in Figure 10b, and the sound speed change in the upper and lower water bodies did not exceed 5 m/s.

**Figure 11:**
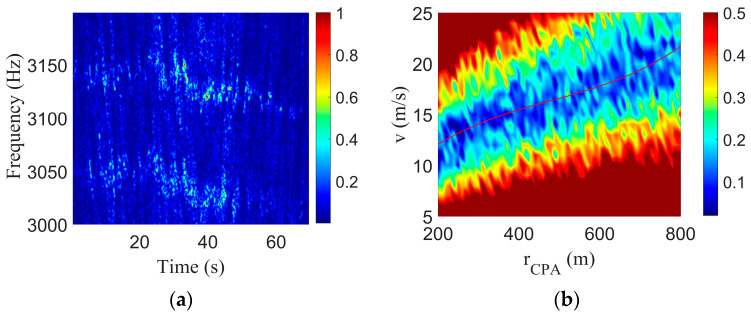
Result of Doppler-warping transformation of the speedboat experiment. (**a**) LOFAR of the signal of speedboat; (**b**) Result of Doppler-warping transformation.

According to the GPS data, the 70 s signal recorded by the hydrophone was selected, the speed of the speedboat was 16 m/s, the range at the closest point of approach was 467 m, and the time of the closest point of approach was 39 s during the signal selection period. The STFT can be utilized in the selected signal to obtain the LOFARgram, as shown in Figure 11a, where the window length was 1 s, the step length was 0.1 s, and the window function type was Hanning. There was a significant Doppler shift phenomenon in the two tones. The signal at approximately 3050 Hz was selected for the Doppler-warping transformation. The sound speed *c* = 1510 m/s, which is the average sound speed of the water body, the speed search grid was *v* = [5:1:25], and the $r_{CPA}$ search grid was [200:10:800]. The search results are shown in Figure 11b, and the red curve in the figure is the parametric coupling curve obtained by cubic polynomial fitting. As is shown in Figure 11a, the instantaneous frequency of the tonal signal was no longer a single frequency but had a certain bandwidth of approximately 20 Hz, the signal was not stable, and there was a certain frequency jitter phenomenon, which would lead to the final estimation error.

**Figure 12:**
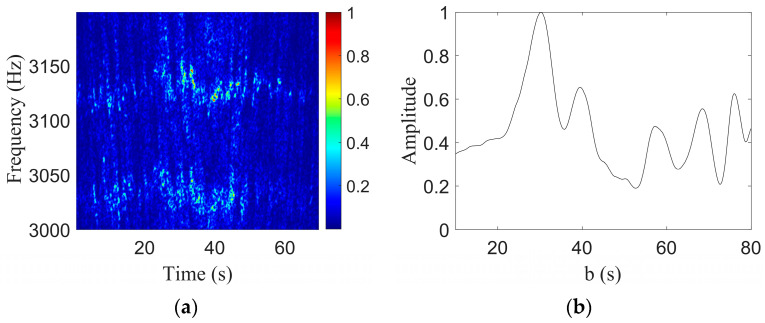
Results of the two-tone interference in the speedboat experiment. (**a**) LOFARgram of the signal without a Doppler shift; (**b**) Search results for b.

The original signal was resampled using the Doppler-warping transformation to eliminate the Doppler shift. Then, the STFT was used in the signal without a Doppler shift to obtain the LOFARgram, as shown in Figure 12a. A comparison with Figure 11a showed that the original Doppler caused the frequency shift to be eliminated, but there were still frequency jitter and band wide problems. In the second step of the method, the two tones in the LOFAR were selected, and the motion parameter b was obtained by the undulating structure of the intensity of the tones, where the waveguide invariant *β* = 0.92 and the b search grid was [10:0.1:80]. The search results are shown in Figure 12b, where the abscissa corresponding to the peak was the final parameter estimate result $\hat{b}=29.4s$.

**Figure 13:**
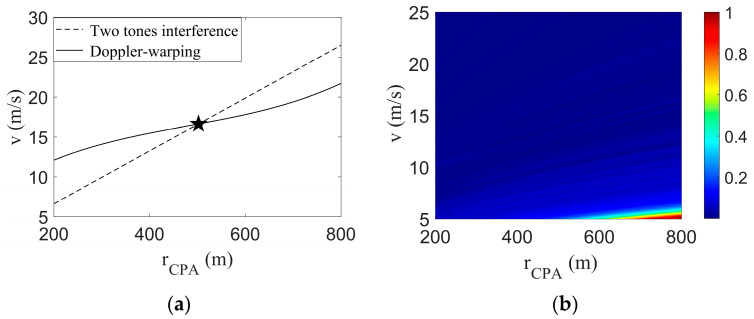
The search results of the speedboat experiment. And the black star represents the position of the parameters estimated by the algorithm. (**a**) The search results of the method proposed in this paper; (**b**) FDM search results.

The parameter coupling curve obtained by the Doppler-warping transformation was combined with the parametric coupling line obtained by the two-tone interference, as shown in Figure 13.

In Figure 13a, the dotted line represents the tone intensity undulating search results, the solid line represents the Doppler-warping search results, the intersection position of the two lines is shown in the five-pointed star, and its corresponding parameter estimates were $\hat{v}=16.41\;m/s$ and ${\hat{r}}_{CPA}=482.4\;m$. The true values of the two parameters were v=16.00 m/s and $r_{CPA}=467\;m$, and the parameter estimation errors were 2.7% and 3.3%, respectively, which were less than 5%.

In the condition of unknown environmental parameters, the waveguide invariant value was set to 1, the sound speed of the water body was 1500 m/s, and the estimation errors of parameters *v* and $r_{CPA}$ were 6% and 15%, respectively.

Figure 13b shows the FDM algorithm search result, and we can see that the algorithm failed at this time. The main reasons are as follows: (1) The signal time was too short, at only 70 s; and (2) The Doppler frequency shift was too large, resulting in the inability to extract frequency information.

## 4. Conclusions

Aimed at the coupling problem of parameters *v* and $r_{CPA}$ when estimating the target motion parameters from tonal signals with a single hydrophone, a method that combines modal interference and the Doppler shift carried by tonal signals was proposed. This method only uses two or more tonal signals radiated by the same target to achieve the estimation of the motion parameters *v* and $r_{CPA}$, and does not require the use of the hydrophone array or the broadband noise radiated by the target. The proposed method fully utilizes the Doppler and modal interference information carried by the tonal signals and only involves the sound speed *c* of the water body and the waveguide invariant *β* information to achieve the target motion parameter estimation. The proposed method requires that the sound source contain at least two tonal signals. The proposed method was applied to the simulation experimental data, and under the condition of a signal-to-noise ratio of 6 dB, the estimation errors of both parameters were less than 10%. Then, the proposed method was applied to the data of two sea experiments. The estimation errors of the two parameters were less than 10%, which shows that the method is effective in terms of its practical application. The method proposed in this paper can be carried out on flexible platforms such as AUVs and buoys, with a low cost. However, it can also be arranged in a large area, so as to achieve target tracking and monitoring of large areas of the sea.

The proposed method can be used in a real-time system to realize the real-time estimation of target velocity and nearest distance information. If there are multiple platforms nearby, the location information of the platform itself and the target motion parameters obtained can be combined to finally achieve the estimation of the target motion trajectory and achieve real-time monitoring and tracking of the target. This also represents the next research direction of the authors of this paper.

## Data Availability

Data can be found at http://swellex96.ucsd.edu (accessed on 13 June 2023).

## References

[1] Arrichiello F., Antonelli G., Aguiar A.P., Pascoal A. (2013). An Observability Metric for Underwater Vehicle Localization Using Range Measurements. Sensors.

[2] Mandic F., Miskovic N., Loncar I. (2020). Underwater acoustic source seeking using time-difference-of-arrival measurements. IEEE J. Ocean. Eng..

[3] Alcocer A., Oliveira P., Pascoal A. (2007). Study and implementation of an EKF GIB-based underwater positioning system. Control Eng. Pract..

[4] Tan Y.T., Gao R., Chitre M. (2014). Cooperative path planning for range-only localization using a single moving beacon. IEEE J. Ocean. Eng..

[5] Gadre A., Stilwell D. Toward underwater navigation based on range measurements from a single location. Proceedings of the IEEE International Conference on Robotics and Automation.

[6] Chen V.C. Analysis of radar micro-Doppler with time-frequency transform. Proceedings of the Tenth IEEE Workshop on Statistical Signal and Array Processing.

[7] Tahmoush D. (2015). Review of micro-Doppler signatures. IET Radar Sonar Navig..

[8] Amiri R., Shahzadi A. (2020). Micro-Doppler based target classification in ground surveillance radar systems. Digit. Signal Prog..

[9] Carter G.C. (1981). Time delay estimation for passive sonar signal processing. IEEE Trans. Acoust. Speech Signal Process..

[10] Nardone S.C., Lindgren A.G., Gong K.F. (1984). Fundamental properties and performance of conventional bearings-only target motion analysis. IEEE Trans. Autom. Control.

[11] Bucker H.P. (1976). Use of Calculated Sound Fields and Matched-field Detection to Locate Sound Sources in Shallow Water. J. Acoust. Soc. Am..

[12] Chuprov S.D., Brekhovskikh L.M., Andreevoi I.B. (1982). Interference structure of a sound field in a layered ocean. Ocean Acoustics, Current State.

[13] Cockrell K.L., Schmidt H. (2010). Robust Passive Range Estimation Using the Waveguide Invariant. J. Acoust. Soc. Am..

[14] Tao H., Hickman G., Krolik J.L. Single hydrophone passive localization of transiting acoustic sources. Proceedings of the OCEANS 2007 Europe.

[15] Turgut A., Orr M., Rouseff D. (2010). Broadband source localization using horizontal-beam acoustic intensity striations. J. Acoust. Soc. Am..

[16] Sun D., Lu M., Mei J., Wang S., Pei Y. (2021). Generalized Radon transform approach to target motion parameter estimation using a stationary underwater vector hydrophone. J. Acoust. Soc. Am..

[17] Sun K., Gao D., Gao D., Song W., Li X. (2023). Estimation of motion parameters of underwater acoustic targets by combining Doppler shift and interference spectrum. Acta Acust..

[18] Arveson P.T., Vendittis D.J. (2000). Radiated noise characteristics of a modern cargo ship. J. Acoust. Soc. Am..

[19] Wang Q., Wan C.R. (2005). A novel CFAR tonal detector using phase compensation. IEEE J. Ocean. Eng..

[20] Rakotonarivo S.T., Kuperman W.A. (2012). Model-Independent Range Localization of a Moving Source in Shallow Water. J. Acoust. Soc. Am..

[21] Young A.H., Harms H.A., Hickman G.W., Rogers J.S., Krolik J.L. (2020). Waveguide-Invariant-Based Ranging and Receiver Localization Using Tonal Sources of Opportunity. IEEE J. Ocean. Eng..

[22] Harms A., Odom J.L., Krolik J.L. Ocean acoustic waveguide invariant parameter estimation using tonal noise sources. Proceedings of the 2015 IEEE International Conference on Acoustics, Speech and Signal Processing (ICASSP).

[23] Chi J., Gao D., Zhang X., Zhang X., Wang Z. (2021). Motion parameter estimation of multitonal sources with a single hydrophone. JASA Express Lett..

[24] Ferguson B.G., Quinn B.G. (1994). Application of the Short-time Fourier Transform and the Wigner–Ville Distribution to the Acoustic Localization of Aircraft. J. Acoust. Soc. Am..

[25] Quinn B.G. (1995). Doppler Speed and Range Estimation Using Frequency and Amplitude Estimates. J. Acoust. Soc. Am..

[26] Xu L., Yang Y., Yu S. (2015). Analysis of moving source characteristics using polynomial chirplet transform. J. Acoust. Soc. Am..

[27] Zou H., Chen Y., Zhu J., Dai Q., Wu G., Li Y. (2004). Steady-motion-based Dopplerlet transform: Application to the estimation of range and speed of a moving sound source. IEEE J. Ocean. Eng..

[28] Sun Q., Wu F.Y., Yang K., Ma Y. (2021). Estimation of multipath delay-Doppler parameters from moving LFM signals in shallow water. Ocean Eng..

[29] Zou N., He J., Shen T., Zou W. Passive Estimation Method For Motion Parameters Of Underwater Near-Field Moving Target. Proceedings of the 2021 OES China Ocean Acoustics (COA).

[30] Gao D.Y., Gao D.Z., Chi J., Wang L., Song W.H. (2021). Doppler-warping transform and its application to estimating acoustic target velocity. Acta Phys. Sin..

[31] Grachev G.A. (1993). Theory of acoustic field invariants in layered waveguides. Acoust. Phys..

